# Prescription Department

**Published:** 1893-08

**Authors:** 


CATARRHAL JAUNDICE.

Dr. N. S. Davis prescribes with success the following :

℞. Hydrarg, chlorid. cor., gr. iv.
Potassii chloridi......gr. cxxviij
Tinct. hyoscyami.....f ℥ iij.
Tinct. cinchonæ......f ℥ v.
M. Sig.—Teaspoonful four times a day.—*Med. Fortnightly.*

SORE THROAT.

The following is recommended in the treatment of sore throat :

℞. Cocainæ hydrochlorat...gr. viij.
Acid. carbolici.........ʒ j.
Glycerinæ..............f ʒ iv.
Aquæ rosæ.....q. s. ad f ℥ xij.
M. Sig.—to be diluted with an equal quantity of water, and used alternately as a spray and gargle. *Med. and Surg. Reporter.*

HAY FEVER.

In a case which appeared to be produced by eating tomatoes, Dr. J. N. Muenich, of Jefferson, Wis., obtained a good result from the use of an ointment composed of

℞. Cocaine muriate........gr. iij.
Thymol................gr. iij.
Bismuth subcarbonate...gr. xij.
Vaselin................℥ j.
M. Sig.—Apply frequently.

DIPHTHERIA.

Dr. S. Cooke Ingraham, of Philadelphia, speaks well of

℞. Ammonii chloridi.......ʒ vj.

Tinct. ferri chloridi.....f ʒ vj.

Acidi hydrochl. dil.......m xxiv.

Hydrarg. chlor. corros...gr. ⅛.

Glycerini...............℥ ss.

Aquæ menth. pip., q. s. ad f ℥ iij.

M. Sig.—A teaspoonful in water every two hours.—*Med. World.*

ANTISEPTIC TREATMENT OF CHRONIC DIARRHŒA.

℞. Acid salicyl...... 1 gramme.
Aque destil.......500 grammes.
Allcohol .........q. s.

M. Sig.—For one rectal injection. This is injected through a long tube. The injection is repeated several times a day.

ALSO :

℞. Salol............. 4 grammes.
Ol. ricini......... 20 grammes.
Syrup rhei....... 40 grammes.
Aquæ cinnamon. .150 grammes.
Gum Arabic......q. s.

M. Sig.—Tablespoonful every hour till the effect is produced.

For diet, milk, eggs, etc., are ordered.

CHRONIC GOUT.

(Dr. A. B. Garrod.)

℞. Ext. colchici acet........gr. vi.
Ext. rhei...............gr. vi.
Ext. aloes socot.........gr. vi.
Ext. belladonnae........gr. i.
Et ft., pil. No. VI.

M. Sig.—Take one at night, twice a week.

FOR REMOVING WARTS.

By Dr. J. Abbott Cantrell.

℞. Acid salicylici.........grs. xxx.
Ungt. aquæ rosæ...... ℥ ss.

M. Sig.—Apply twice daily for two days, after which the growths being softened, they should be removed by a dermal curette, and by using these means you can safely say that the wart will not return.

SYPHILIS.

℞. (*Liquor of Van Swieten.*)
Hydrarg. bichloridi....1 part.
Alcohol............100 pts.
Aquæ..............900 pts. M.
Sig.—Teaspoonful after each meal.

